# Absorption in Sport: A Cross-Validation Study

**DOI:** 10.3389/fpsyg.2017.01419

**Published:** 2017-08-22

**Authors:** Stefan Koehn, Nektarios A. M. Stavrou, Jeremy Cogley, Tony Morris, Erez Mosek, Anthony P. Watt

**Affiliations:** ^1^School of Health Sciences, Liverpool Hope University Liverpool, United Kingdom; ^2^Faculty of Physical Education and Sport Science, National and Kapodistrian, University of Athens Athens, Greece; ^3^Sport Psychology Department, Hellenic Sports Research Institute, Olympic Athletic Center of Athens “Spiros Louis” Athens, Greece; ^4^College of Sport and Exercise Science, Victoria University of Technology Melbourne, Melbourne VIC, Australia

**Keywords:** absorption, flow, cross-validation, reliability, validity

## Abstract

Absorption has been identified as readiness for experiences of deep involvement in the task. Conceptually, absorption is a key psychological construct, incorporating experiential, cognitive, and motivational components. Although, no operationalization of the construct has been provided to facilitate research in this area, the purpose of this research was the development and examination of the psychometric properties of a sport-specific measure of absorption that evolved from the use of the modified Tellegen Absorption Scale (MODTAS; Jamieson, 2005) in mainstream psychology. The study aimed to provide evidence of the psychometric properties, reliability, and validity of the Measure of Absorption in Sport Contexts (MASCs). The psychometric examination included a calibration sample from Scotland and a cross-validation sample from Australia using a cross-sectional design. The item pool was developed based on existing items from the modified Tellegen Absorption Scale (Jamieson, 2005). The MODTAS items were reworded and translated into a sport context. The Scottish sample consisted of 292 participants and the Australian sample of 314 participants. Congeneric model testing and confirmatory factor analysis for both samples and multi-group invariance testing across samples was used. In the cross-validation sample the MASC subscales showed acceptable internal consistency and construct reliability (≥0.70). Excellent fit indices were found for the final 18-item, six-factor measure in the cross-validation sample, $\chi_{\left(120\right)}^{2}$ = 197.486, *p* < 0.001; CFI = 0.957; TLI = 0.945; RMSEA = 0.045; SRMR = 0.044. Multi-group invariance testing revealed no differences in item meaning, except for two items. The MASC and the Dispositional Flow Scale-2 showed moderate-to-strong positive correlations in both samples, *r* = 0.38, *p* < 0.001 and *r* = 0.42, *p* < 0.001, supporting the external validity of the MASC. This article provides initial evidence in support of the psychometric properties, reliability, and validity of the sport-specific measure of absorption. The MASC provides rich research opportunities in sport psychology that can enhance the theoretical understanding between absorption and related constructs and facilitate future intervention studies.

## Introduction

Originating in mainstream psychology, absorption reflects a readiness for experiences of deep involvement in the task (Tellegen and Atkinson, 1974). Tellegen and Atkinson (1974) defined absorption as “a disposition for having episodes of ‘total’ attention” that “result in a heightened sense of the reality of the attentional object, impervious to distracting events […] including an emphatically altered sense of self” (p. 268). Roche and McConkey (1990) described absorption as a characteristic that involves openness to experience of emotional and cognitive alterations across a variety of situations. Tellegen and Atkinson (1974) developed a multi-dimensional self-report instrument, named the Tellegen Absorption Scale (TAS), as a trait measure to examine inter-individual differences in people’s tendency to experience altered states of consciousness. Additionally, absorption was found to be associated with hypnotic susceptibility (Tellegen and Atkinson, 1974) and imagery (Roche and McConkey, 1990). In sport psychology, absorption has rarely been empirically addressed, which is mainly due to the lack of an adequate scale that provides the opportunity to operationalize and analyze research objectives. The purpose of the present study was the development and validation of a parsimonious, sport-specific measure of absorption, based on prior work on a generic measure of absorption.

Within the framework of absorption, Tellegen (1981) proposed two conceptually different modes of functioning, characterized by an instrumental set and an experiential set. The instrumental set involves “active, realistic, voluntary and relatively effortful planning, and decision making and goal directed behavior” (Tellegen, 1981, p. 222), whereas the experiential set reflects an involuntary, effortless and deep involvement in task. Individuals prone to absorption are more likely to adopt an experiential rather than an instrumental mode of functioning, particularly in situations with task relevant stimuli that encourages experiential activity (Tellegen, 1981). A conceptual link between absorption and flow theory is evident in the description of experimental and instrumental sets of functioning and flow experiences referred to as ‘letting it happen’ and ‘making it happen’ (Swann et al., 2016). The experiential set corresponds with feelings of effortlessness and automaticity frequently experienced during flow. Instrumental modes of functioning as well as states referred to as ‘making it happen’ are signified by a heightened sense of concentration, effortfulness, situation appraisals, and goal-directed behavior. The conceptual association between absorption and flow forms the basis for testing the external validity of the Measure of Absorption in Sport Context (MASC) in conjunction with flow.

A review on absorption and related constructs, such as suggestibility and hypnotizability, revealed rather low correlations between these constructs (Roche and McConkey, 1990). Sapp (2015) suggests that the reason for such low correlations is likely due to the absence of purposive sampling, including the recruitment of participants who are not susceptible to measurements of the intended effect. Similarly from a conceptual perspective, Tellegen (1992, Unpublished) proposed that highly absorbed individuals experience alterations in their consciousness which is reflected in an expansion of their cognition, awareness, reminiscence, a narrowing in engaging stimuli, dissociative involvement, and synesthetic experience. Evidence for individual differences on absorption and different ways of functioning has been provided by Qualls and Sheehan (1981). Applying instructional and experiential conditions to different absorption groups, Qualls and Sheehan showed that participants scoring low on absorption achieved deep relaxation when they followed a goal-directed set of instructions on external stimuli (i.e., instrumental mode of functioning), whereas high-absorbers preferred an internal, imagery-based approach that led to deep relaxation (i.e., experiential mode of functioning). The results indicated that absorption is a potential moderating factor and in conjunction with using relaxation techniques, this is a method that is frequently used to achieve better performance and deeper flow experiences in sports (e.g., Pates and Maynard, 2000; Koehn et al., 2014).

As an operationalization of absorption, Tellegen and Atkinson’s (1974) instrument evaluated different aspects of absorption, such as responsiveness to engaging stimuli, synesthesia, enhanced cognition, dissociative involvement, vivid reminiscence, and enhanced awareness. Responsiveness to engaging stimuli reflects a strong emotional response to the beauty found in art or nature (Ott, 2007). Synesthesia means ‘joint sensation,’ stemming from the Greek word anesthesia (no sensation), reflecting the use of cross-modal associations; for instance sensory input in one modality, such as a number or symbol, is experienced in a different modality, such as color (Nikolic et al., 2011). Enhanced cognition indicates people’s extra-sensory perception, which is associated with imaginational thinking (Ott, 2007). Dissociative involvement refers to oblivious experiences, for instance when individuals get lost in listening to a song or watching a movie. Masters and Ogles (1998) described dissociation in pathological terms and, more importantly in sport psychological context, as changes in attentional focus. An internal focus of attention relates to people’s perceptions and physical sensation, whereas an external focus relates to information outside of the body, and is referred to as dissociation. Nideffer’s (1986) theoretical framework on attentional focus has addressed the concept of dissociation in sport. Another dissociative strategy that may affect absorption is reflected in Brick et al.’s (2015) work on metacognitive processes. In a study with endurance runners, Brick et al. (2015) examined different dissociative strategies and how runners deal with contextual needs. For instance, during longer performance tasks athletes engaged in active distraction and letting their mind wander to deal with various aspects, such as boredom. On the other hand, when running felt hard athletes actively engaged in self-regulatory process, such as use of relaxation techniques, self-talk, and mindfulness techniques. Vivid reminiscence characterizes the vivid recollection and use of past experiences in a present context (Tellegen and Atkinson, 1974). Enhanced awareness is associated with peak experiences, in which the individuals thinking evolves to a higher level and processed information appears more meaningful (Ott, 2007). In sum, enhanced absorption would provide a lower potential for distraction and a higher potential for meaningful involvement in the task at hand (Tellegen and Atkinson, 1974; Masters and Ogles, 1998; Ott, 2007; Ward, 2013).

Absorption in sport has been mainly discussed as an umbrella term to explain sport phenomena on a conceptual level without the support of empirical evidence (Privette, 1983; Jackson, 2000). Privette (1983) and Jackson (2000) provided a conceptual overview of positive experiential states, which identified absorption as one of the communal aspects that is shared by peak performance, peak experience, and flow. Privette (1983) proposed that peak experiences do not necessarily arise as a result of participation in a specific activity. Individuals could be in a passive mode, which may occur in inactive or non-motivated states in everyday life, for instance, by listening to radio or music, watching television, or forms of intoxication. In contrast, flow experience and peak performance reflect the active nature of the physical or mental immersion in planned and structured activities as characterized by cognitive involvement, interactivity, and responsiveness between athletes and their environments (Privette, 1983). More recently, a research group around Swann et al. (2016, 2017) proposed a flow concept linking flow state with performance and absorption. This framework also emphasizes the active nature when athletes are in a flow state. Similarly, a sport-specific measure of absorption would need to reflect the active involvement of athletes in their activities.

There is a discrepancy between the conceptual discussion on absorption and the operational measurement testing absorption in sport. Researchers frequently referred to absorption when defining flow experiences (e.g., Jackson, 1995; Jackson et al., 2008; Martin and Jackson, 2008), although little research has been conducted on the interplay of the two constructs. Martin and Jackson’s (2008) examined task absorption in various activities, such as music, work and sport. The measurement of absorption was conducted through a nine-item flow short form (Jackson et al., 2008), reflecting the nine dimensions of flow (challenge-skills balance, action-awareness merging, clear goals, unambiguous feedback, concentration on the task at hand, sense of control, loss of self-consciousness, time transformation, autotelic experience) and a 10-item ‘core’ flow measure, whose items ask whether participants are “totally involved,” “totally focused,” “tuned in,” or “in the zone” (p. 155). Although the authors’ aimed to examine task absorption through the assessment of two brief flow measures, the used measures were an operationalization of flow rather than specific measures to examine absorption. Martin and Jackson (2008) proposed that the use of one questionnaire over the other should be dictated by study purpose and question. One limitation of this study was that the conceptual and operational link of the examination of task absorption is not particularly strong, for instance equating task absorption with flow may not take into account unique aspects of absorption that are not reflected in flow, such as the merging of various senses into a unified experience or higher cognitive processes. In fact, Martin and Jackson (2008) found a positive relationship between the two measures, but only about half of the variance was explained, indicating that both scales measure distinctly different constructs, serving “different operational purposes” (p. 152). Absorption has been viewed as a complex, multi-dimensional construct with a trait dimension (Tellegen and Atkinson, 1974; Sapp, 2015), and future research needs to provide clear conceptual background and operational purpose of a sport-specific absorption measure.

One of the few studies in sport psychology using the TAS has been conducted by Koehn et al. (2013). The TAS was correlated with flow [measured through the Dispositional Flow Scale-2 (DFS-2); Jackson and Eklund, 2002], confidence [measured through the Trait Sport Confidence Inventory (TSCI); Vealey, 1986], and imagery use [measured through the Sport Imagery Questionnaire (SIQ); Hall et al., 1998]. The results indicated close to zero correlations between absorption and dispositional flow (*r* = 0.02; *ns*) and confidence (*r* = 0.00; *ns*), whereas a small, positive relationship emerged between absorption and imagery use (*r* = 0.20; *p* < 0.01). The findings suggested that absorption is orthogonal to flow and confidence and might vary independently from these constructs.

Koehn et al. (2013) pointed out that the contents of the TAS items could have had a major influence on the results, as the TAS assesses absorption in various non-sport-specific contexts, such as poetic language, music, art, and nature. Furthermore, activities described in the TAS, such as watching TV or listening to music, are rather passive activities, whereas sport activities are signified by dynamic physical and mental involvement. A sport-specific version of absorption could provide more conclusive evidence on the relationship between absorption and flow. More importantly, additional research would help in the investigation of conceptually related constructs, facilitating the advancement of theoretical models and interventions in sport.

The aims of the cross-validation study were threefold, including (a) item development, (b) assessments of internal consistency and validity of the MASC in the calibration sample (CS), and (c) the re-examination of the results in a separate validation sample (VS). It was hypothesized that the measurement model of the MASC would corroborate the original six-factor structure of absorption in both samples, as modifications had been implemented on an item level (i.e., MODTAS; Jamieson, 2005) but not on a factor level (H1). Configural and structural invariance and the invariance of latent mean structures are expected to be found across samples (H2). Positive relationships are expected between absorption and flow, providing evidence for the external validity of the MASC (H3).

## Materials and Methods

### Participants

A total of 606 male (*n* = 361) and female (*n* = 245) athletes between 16 and 48 years of age participated in the MASC development and assessment phases. One CS from Scotland and one VS from Australia were employed. The CS consisted of 292 University students (*n*_males_ = 195; *n*_females_ = 97). The majority of participants played soccer (*n* = 109), rugby (*n* = 34), basketball (*n* = 19), or hockey (*n* = 19). The age of this sample ranged from 16 to 32 years (*M* = 20.00; *SD* = 2.41). The VS consisted of 314 (*n*_males_ = 166; *n*_females_ = 148) physical education and sport psychology students from a major city in Australia. The age of the sample ranged from 17 to 48 years (*M* = 20.65; *SD* = 3.62), with a majority of participants involved in Australian Rules Football (*n* = 74), netball (*n* = 51), basketball (*n* = 42), and soccer (*n* = 23). Sports experience ranged between 2 and 19 years (*M* = 9.66; *SD* = 4.84).

### Measures

#### Measure of Absorption in Sport Contexts (MASCs)

The MASC was developed to assess athletes’ perception to get absorbed into sport situations. Instead of using the original true-false response format (Tellegen and Atkinson, 1974), a frequency-based Likert format was preferred based on successful TAS item-response modifications (Jamieson, 2005). Jamieson (2005) argued that a binary response format would under-represent item variance and, potentially, the covariance structure. The response scale is a seven-point Likert-type scale anchored by 1 (*strongly disagree*) and 7 (*strongly agree*). The seven-point response format was chosen to facilitate the examination of measurement models. Rhemtulla et al. (2012) provided evidence that the use of 6–7 categories is preferable when using maximum likelihood methodologies.

#### Dispositional Flow Scale-2

The DFS-2 (Jackson and Eklund, 2002) consists of 36 items representing nine subscales, each comprising four items. The response format is a five-point Likert scale anchored by 1 (*never*) and 5 (*always*), assessing respondents’ frequency of flow experiences. The subscales showed acceptable reliability values, ranging between 0.81 and 0.90 (Jackson and Eklund, 2002). The DFS-2 questionnaire was identical in both samples.

### Procedures

Following approval by the Universities’ Ethics Committees, we requested access to students with a sport background who took part in sport psychology and physical education classes. Standard consent procedures were followed before questionnaire administration. The participants filled in the demographic, absorption, and flow measures within their organizational setting at the University. Participants completed the questionnaire battery within 15 min. The procedures were identical across samples.

This study was carried out in accordance with the recommendations of name of guidelines, name of committee, with written informed consent from all subjects. All subjects gave written informed consent in accordance with the Declaration of Helsinki. The protocol was approved by the Universities’ (Victoria University, Melbourne; University of Abertay, Scotland) Ethics Committees.

### Statistical Analysis

Confirmatory factor analysis (CFA) was undertaken using AMOS 20.0 software. Model estimations were based on maximum likelihood methods (Arbuckle, 2012). In order to establish and assess multi-factor measurement models we adopted a strategy by Jöreskog (1993), who proposed a model-generating stage before testing the measurement model. First, we assessed one-factor congeneric models for each of the six MASC factors. Congeneric models are the smallest entity of a measurement model outlining the regression of a number of observed variables (items) on a single latent variable (factor). This allows re-specification of the model (i.e., freeing covariance, item deletion) before calculating the overall measurement model. Following Jöreskog’s (1993) data analysis strategy, the established measurement model will then be tested twice with a calibration and a VS. The measurement models were assessed through parsimonious fit, we employed a chi-square test (χ^2^), incremental fit was examined through the comparative fit index (CFI; Bentler, 1990) and the Tucker-Lewis index (TLI; Bollen, 1989), and absolute fit was analyzed through the standardized root-mean-square residual (SRMR; Hu and Bentler, 1998) and the root-mean-square error of approximation (RMSEA; Steiger, 1990). Based on the standards developed by Hu and Bentler (1999), a very good fit of the data is achieved when CFI and TLI scores exceed 0.95, SRMR is below 0.08, and RMSEA is below 0.06. The statistical analyzes were identical for CS and VS.

### Item Development

The MODTAS (Jamieson, 2005) is a self-report measure, consisting of 34 items and six subscales. We developed the item pool on the MODTAS items, that is, we re-wrote the contents of the MODTAS items from an everyday context and transferred into a sport-specific context. Examples of original items for each of the six TAS subscales (equivalent items in a sport-specific are displayed in Supplementary Table 5) are for Factor 1: “I can be greatly moved by eloquent or poetic language” (responsive to engaging stimuli, see Item 1 in Supplementary Table 5); Factor 2: “The sound of a voice can be so fascinating to me that I can just go on listening to it” (synesthesia, see Item 6 in Supplementary Table 5); Factor 3: “I often have ‘physical memories’; for example, after I’ve been swimming, I may still feel as if I’m in the water” (enhanced cognition, see Item 7 in Supplementary Table 5); Factor 4: “It is sometimes possible for me to be completely immersed in nature or in art and to feel as if my whole state of consciousness has somehow been temporarily altered” (oblivious/dissociative involvement, see Item 11 in Supplementary Table 5); Factor 5: “I can sometimes recollect certain past experiences in my life with such clarity and vividness that it is like living them again or almost so” (vivid reminiscence, see Item 15 in Supplementary Table 5); and Factor 6: “I sometimes ‘step outside’ my usual self and experience an entirely different state of being” (enhanced awareness, see Item 16 in Supplementary Table 5). For some of the MODTAS items, e.g., “Sometimes I can change noise into music by the way I listen to it,” it was difficult to develop a sport-specific equivalent. In total, six items were omitted from the study. To assure item content validity, two experts with relevant experience in the field reviewed the wording of the items and provided feedback on potential issues and item modifications in a content validity analysis. Following discussion on item suitability, the authors reached a consensus on the inclusion, revision, or rejection of each item in the item pool. Based on the 34-item MODTAS (Jamieson, 2005), we developed the sport-specific MASC scale, consisting of 28 items and six factors (five items for Factors 1, 2, and 3, respectively; six items for Factor 4; three items for Factor 5; four items for Factor 6).

## Results

### Reliability

Cronbach’s alpha showed adequate values for most MASC subscales in the CS, ranging from 0.65 (vivid reminiscence) to 0.78 (dissociate involvement). The construct reliability examines the internal consistency of a set of measures, whereas the variance extracted estimates indicate the total amount of variance in the indicators accounted for by the latent construct (Bollen, 1989). In order to calculate construct reliability, ρ_η_, and the variance extracted estimate, ρ_vc(η)_, the formula by Fornell and Larcker (1981) was used. The lower acceptable values for reliability composites should be 0.70 (Yi and Davis, 2003). Although ρ_η_ and ρ_vc(η)_ are based on calculations of the overall measurement model, in the interest of brevity the results are shown in Supplementary Table 1. Model-based reliability estimates were above 0.70 except vivid reminiscence (0.64). For the VS, Cronbach’s alphas for all subscales were acceptable, ranging between 0.70 (enhanced cognition) and 0.80 (synesthesia). The construct reliability was tenable, all ρ_η_ values were at 0.70 or higher (Supplementary Table 1).

### Convergent Validity

In the calibration phase, all MASC items showed adequate factor loadings between 0.39 and 0.79 (*M*_loadings_ = 0.61). The estimated parameters were significantly different from zero, providing support for the MASC’s convergent validity. The unidimensional constructs were examined through the fit indices shown in Supplementary Table 1. Except for synesthesia and enhanced cognition, the CS congeneric models showed a very good fit of the data with CFI and TLI values of 0.95 and above. The congeneric model capturing dissociative involvement with six indicator items needed to be re-specified as several indices showed poor fit of the data (χ^2^ = 73.872; *p* < 0.001; CFI = 0.867; TLI = 0.779; RMSEA = 0.157; SRMR = 0.070). Even though all regression weights were significant, standardized residual covariances above 2.0 were found for two items. Modification indices and critical ratios indicated that item removal would lead to a substantial improvement of the model. Following elimination of both items the results showed acceptable fit indices for dissociative involvement (Supplementary Table 1).

In the validation phase, the factor loadings showed acceptable scores for each congeneric model, ranging between 0.39 and 0.86, with an average of *M*_loadings_ = 0.63. The construct validity showed a good fit of the data, with all indices showing acceptable fit (Supplementary Table 1). The results for the VS were generally stronger than for the CS. Only two congeneric models revealed significant *p*-values, indicating less optimal fit. Synesthesia was the only factor that indicated a weaker fit of the data across samples.

### Hierarchical Confirmatory Factor Analysis

When examining multi-group invariance, Byrne (2004, 2010) proposed a testing strategy that involves a number of hierarchical steps. Firstly, a baseline model needs to be established for each sample. Byrne suggested that testing should be guided by best fit of the data and model parsimony.

The calibration measurement of the MASC included 26 items. The results showed a poor fit of the data for the single-factor model, the second-order model, and the six-factor uncorrelated and correlated models. The correlated model reflecting the original hypothesized six-factor structure and the best fit of the data was, therefore, used for further analyzes (Supplementary Table 2). A number of items in the six-factor correlated measurement model appeared to be problematic. One item (synesthesia) cross-loaded onto the dissociative involvement factor and was subsequently deleted. Modification indices for regression weights with scores above 10 and covariances above 4 (Lagrange Multiplier test for standardized residual covariances) indicated poor fit for up to seven items. The final and most parsimonious model (Model 4b) indicated a very good fit of the data, $\chi_{\left(120\right)}^{2}$ = 207.196, *p* < 0.001; CFI = 0.941; TLI = 0.925; RMSEA = 0.050; and SRMR = 0.050. All regression coefficients were significant, ranging between 0.42 and 0.90, supporting the validity of the item-factor relationship (with three items per factor). Testing the VS provided similar results to the initial assessment. Following model modifications using the same procedures in both samples, the final 18-item, correlated six-factor model (M4b) showed appropriate fit values of the tested models (Supplementary Table 2). The incremental fit indices CFI and TLI were slightly stronger than the results of the CS with values around the 0.95 level, indicating a very good fit of the data.

### Testing Measurement Invariance of the 18-Item Six-Factor Model

Assessing measurement invariance with multi-group CFA, Gregorich (2006) proposed a hierarchical approach to the testing procedure, including the comparison of three models assessing the (i) metric invariance (factor loadings constrained to be invariant), (ii) strong invariance (factor loadings and item intercepts constrained to be invariant) and (iii) strict invariance (factor loadings, item intercepts, and item variance) against a configural model that is freely estimated with no constraints. Support for metric invariance will indicate whether factors have the same meaning across national samples, strong invariance indicates whether comparisons between latent mean differences will be meaningful and strict invariance taking into consideration item residual invariance, providing an even stronger basis for cross-cultural comparisons (Gregorich, 2006).

The results between configural and metric models (Supplementary Table 3) for the group comparison showed acceptable values for the group comparison, ΔCFI < 0.01 (Cheung and Rensvold, 2002). Gregorich (2006) stated that this comparison is particularly important as it indicates whether items have the same meaning across groups. A shortcoming of this technique is that it only allows examination by comparing all the factor loadings across groups, but does not provide pinpoint accuracy when it comes to item comparisons for specific subscales across groups. Although Cheung and Rensvold (2002) stated that the measurement model is completely invariant to the configural model when ΔCFI is less than 0.01, Byrne (2010) considered Δχ^2^ and Δ*df* in conjunction with statistical significance as more stringent. More importantly, in order to show non-invariance testing proceeds on to a subscale level, this way the results allow to pinpoint which items are invariant and non-invariant across groups (Byrne, 2010). With regard to the data, the comparison between the configural model (M1) and the metric model (M2) did no show substantial differences on a factor-loading level, ΔCFI = 0.009, indicating that factor loadings are generally invariant across groups.

Following the test of the metric model, which had all factor loadings constrained, Byrne (2010) suggested to constrain the items of the first latent variable, in this case items of the *responsiveness to engaging stimuli* (RES) factor, equal across groups and to measure the remaining items freely for each sample. Therefore, when “factor-loading parameters are found to be invariant across groups, their specified equality constraints are maintained, cumulatively, throughout the remainder of the invariance-testing process” (p. 223). This procedure is shown in Supplementary Table 3 (see models 5 to 13). Constraining only the responsiveness to engaging stimuli items, there was no significant difference with the configural model, *p* < 0.05 (see model comparison 3 vs. 1). Constraining RES and synesthesia items, the measurement model showed significant differences compared to the configural model, *p* < 0.05 (see model comparison 4 vs. 1). By freely estimating Item 5 of the synesthesia factor, the model comparison between M5 and M1 revealed no significant differences. The same procedure was followed for each of the absorption subscales. Items showing significant invariance across groups included Item 5 (“Some of my most vivid memories of my sport are called to my mind by scents and smells”) and Item 11 (dissociative involvement; “While participating in my sport, it is sometimes possible for me to be completely immersed in the situation and to feel as if my whole state of consciousness has somehow been temporarily altered”). The final measurement model, M10 vs. M1, with 16 constrained items and two freely estimated items, was not significantly different to the configural model, ΔCFI = 0.004.

Testing for strong invariance by constraining factor loadings and item intercepts of the model, the results showed strong support for the model comparison between calibration and validation data, ΔCFI = 0.010, in accordance with Cheung and Rensvold’s (2002) suggestions (ΔCFI < 0.01). Gregorich (2006) proposed that the equivalence of the factor item loadings and item intercepts is a precondition for testing latent mean differences, as applied by Vlachopoulos et al. (2013). The current results, M11 vs. M1, indicated that it would be appropriate to proceed with this type of analysis to compare mean differences in absorption factors between samples, albeit the ΔCFI was close to the 0.01 cut-off. Final model comparisons indicated substantial differences between M12 and M1 and between M13 and M1.

### Latent Mean Differences

Testing for differences in latent factor means, the factor loadings and observed variable intercepts needed to be constrained to be equal across groups. The critical ratio (C.R.), values > ± 1.96, showed no significant differences between groups for five of the six latent factor means. The results were not significant for responsive to engaging stimuli (C.R. = 0.792), synesthesia (C.R. = 0.866), enhanced cognition (C.R. = 1.732), vivid reminiscence (C.R. = 0.177), and enhanced awareness (C.R. = -0.206). One significant result was found for dissociative involvement (C.R. = 2.247; *p* < 0.05). The results supported the invariance of the factor loadings and latent mean structures between groups for the six-factor model (Supplementary Table 4). The 18 items, with three items per factor, showed adequate factor loadings between 0.45 and 0.84 (*M*_loadings_ = 0.67), and item uniqueness between 0.20 and 0.71 (Supplementary Table 5).

### Criterion Validity

The DFS-2 revealed acceptable Cronbach’s alpha values between 0.75 and 0.85 in the CS. On a global level, the correlation coefficient between absorption and flow was moderate-to-strong for the calibration, *r* = 0.38, *p* < 0.001 and the validation, *r* = 0.42, *p* < 0.001, samples. On a subscale level, the majority of correlations were significant at a 0.01 level, showing the expected positive links between the MASC and the DFS-2. Nearly all MASC subscales correlated significantly with flow subscales, although synesthesia showed a number of statistically weaker relationships (Supplementary Table 6).

## Discussion

The purpose of this study was the development and validation of a parsimonious sport-specific version of absorption. The final measurement model for 18 items and six factors indicated very good fit for both calibration and VSs. With regard to model parsimony, the 18-item model was superior to the 26-item model (Supplementary Table 2) and accounted for a larger amount of variance by the latent construct. The construct of absorption is not represented best by a single latent construct, but through its multi-dimensional six-factor structure, confirming the original subscale structure by Tellegen and Atkinson (1974). The final 18-item, six-factor measurement model in the VS showed acceptable reliability with regard to internal consistency and construct reliability, as well as acceptable convergent and criterion validity.

Across both samples we found that the six-factor correlated model reflected a better fit of the data (i.e., comparative and parsimonious fit indices) than other models tested which supported H1. The model comparison showed no significant differences across samples, except on a residual level, providing partial support for H2. Furthermore, latent mean structures revealed that the testing of the six-factor correlated model holds up against two different samples. The findings are encouraging and can be generalized across CS and VS, indicating that both groups operated under a similar conceptual frame of reference. The final first-order six-factor model will be particularly helpful for future research to examine correlates, antecedents and consequences of absorption in sport. Strong support was found for the criterion validity of the new absorption measure. Previous research suggested an orthogonal relationship between absorption and flow (Koehn et al., 2013). In this study we found a significant relationship between the MASC and the DFS-2 supporting H3 and the general theoretical contentions of a positive absorption-flow relationship. On a subscale level synesthesia was the only factor that showed weak correlations with flow dimensions. Synesthetic experience could be more relevant for specific sports in which sounds are an integral part of the performance, or sports that involve musical presentations, such as figure skating or dance that also have relevance for performance. Scent and smells, however, could have less relevance overall and the results in Supplementary Table 3 showed that Item 5 (‘Some of my most vivid memories of my sport are called to mind by scents and smells’) had been interpreted differently between participants of the two samples. It might be that, in a sport context, individuals do not necessarily associate or put a strong emphasis on sounds or smells, which may or may not be part of their performance. An alternative type of synesthesia could be that performances or different performance aspects are related to various colors, as shown by swimming performance and synesthetic color (Nikolic et al., 2011). In the original item pool there was only one item reflecting color synesthesia. This item performed poorly in the CFA and was subsequently deleted.

The results indicated that in contrast to conceptual interpretations (see Privette, 1983), absorption should not be viewed as a unidimensional construct. The findings provided evidence of a multi-dimensional structure underlying absorption in sport. The six-factor structure of the MASC has been re-validated across samples. Latent mean differences between calibration and VSs were only found for the absorption subscale of dissociative involvement. The largest sub-sample from Australia was recruited from the sport of Australian Rules Football (AFL). In comparison to rugby and other team sports, AFL appears to be a rather physical and injury prone sport. In contrast to the Scottish sample, it is possible that these aspects may lead to lower levels of dissociative involvement in AFL athletes. These cultural and sport-related differences may have contributed to variations in item meaning (Supplementary Table 3, Item 11). From a theoretical perspective, more collaborative effort is required to make advancements in the conceptualization of absorption in sport. For instance, an absorption model should reflect the multi-dimensionality of the construct in conjunction with performance and potential correlates.

A number of absorption factors, such as responsiveness to engaging stimuli, dissociative involvement and vivid reminiscence, include items that reflect athletes’ ability to image or stimulate imagination (e.g., Item 2, Supplementary Table 5). Given the conceptual association and research findings between absorption and imagery, athletes high in absorption might have a proneness or ability to using imagery more effectively. Using the absorption measure in the selection process could help practitioners to match participants with optimal intervention strategies. From an applied perspective, based on the findings of this study, athletes who achieve states of high absorption should be better equipped to experience flow, a finding which has relevance for intervention research. Particularly, the implementation and effectiveness of intervention by using imagery as a vehicle to magnify experiential states would provide an interesting avenue for future applied research to improve athlete performance. Practitioners and sport psychologists need to evaluate differences between high- and low-absorbing athletes and potential effects on experiential, cognitive, and performance variables in sport.

There were two main limitations to this study. Firstly, the two samples displayed homogeneity with regard to age. The majority of participants were between 18 and 22 years old. Future studies would do well to strive for greater participant diversity. Using a sample with a broader age range would add to the validity of the current results. Secondly, there might be other aspects to absorption that are currently not included in the six-factor structure. Additional characteristics of absorption could reflect higher cognitive processes in sport, such as anticipation, which were not accounted for in the original version of the TAS particularly in interactive team games (e.g., football, rugby) and individual ball sports (e.g., tennis) or combat (e.g., boxing, judo) sports, where the ability to anticipate opponents’ actions, as well as those of teammates in team games, could also play a relevant part of competition performance and would reflect athletes’ capability to perform advanced cognitive processes.

### Theoretical Significance and Practical Implications

The results indicated that in contrast to conceptual interpretations (Privette, 1983; Jackson, 2000), absorption should not be viewed as a unidimensional construct. Testing the external validity, a single latent factor of absorption does relate to flow on a global and subscale level. Although this finding supports a single factor of absorption, future research would benefit from examining absorption on a more specific and detailed subscale level reflecting all dimensions. The findings provided evidence of a multi-dimensional structure underlying absorption in sport. The results also provide some support for the conceptual links that have been proposed between absorption and various flow dimensions including challenge-skills balance and concentration on the task at hand (Jackson, 2000). The current findings also supported Martin and Jackson’s (2008) conceptual argument regarding an association between flow and absorption. The results of this study indicated that trait absorption in sport and dispositional flow share a small amount of variance, with about 15% explained in the CS and about 18% explained in VS. This indicates that despite some similarities both constructs showed large conceptual differences. These findings are in line with previous results indicating low-to-moderate correlations between absorption, as measured by the TAS, and related constructs of suggestibility and hypnotizability (Roche and McConkey, 1990).

The lack of strong correlations between absorption and associated constructs may, in part, be related to individual differences and whether individuals adopt an instrumental or experiential mode of functioning in the respective activities (Tellegen, 1981). The nature of absorption is yet to be researched in more depth and may depend on a number of cognitive, affective and situational factors (Nandon et al., 1991). For instance, differences could be found on a motivational and cognitive level. Koehn et al. (2016) showed that cognitive imagery ability partially mediated the relationship between cognitive imagery functions and flow, whereas a full mediation model was found between motivational imagery ability, motivational imagery functions and flow. Flow experiences seem to be characterized by motivational and cognitive processes. Neurological research on flow state indicated that different cognitive functions impact on flow. Dietrich (2004) argued that athletes experience flow when using implicit knowledge while the analytical, conscious explicit system is suppressed. This suppression takes place through a state of transient hypofrontatlity, lowering the activity of the explicit system facilitating flow (Dietrich, 2004). This characteristic of flow can be found when athletes have difficulties describing what they did or how they performed during flow. From this perspective, flow reflects a state of expertise induced amnesia (Beilock and Carr, 2001). The conceptual link between cognition (i.e., engaging stimuli, synesthesia, enhanced cognition, dissociative involvement) and individuals’ experiential mode to get absorbed into an activity warrants more specific experimental research. From a theoretical perspective, more collaborative effort is required to make advancements in the conceptualization of absorption in sport. For instance, a sport-specific absorption model should reflect the multi-dimensionality of the construct in conjunction with performance and potential correlates.

From an applied perspective, athletes who achieve states of high absorption should be better equipped to experience flow, which would be relevant for intervention research; particularly in the evaluation of psychological interventions which attempt to use imagery as a potential vehicle to magnify experiential states with the goal of enhancing athletic performance. Practitioners and sport psychologists need to evaluate the potential effects that high- and low-absorption traits have on experiential, cognitive and performance variables in sport.

## Conclusion

Previous discussions addressed absorption as a unidimensional construct (Privette, 1983; Jackson, 2000), but support has been found for the multi-dimensional structure of absorption as evidenced by validity and model tests. In addition, the predictive validity of the MASC needs to be examined in more detail for a range of constructs, including imagery, confidence and motivation. Particularly, the links between absorption and performance should be at the center of investigations. It has also been conceived that absorption entails key experiential, motivational, and cognitive components (Wild et al., 1995); empirical evidence is now needed to develop the theoretical contentions for the role of absorption in various processes in sport. The MASC provides rich opportunities for such research and may help facilitate further conceptual and practical developments in sport psychology.

## Author Contributions

SK: writing and contribution of all parts of the paper, data analysis; NS: writing of discussion, contribution to data analysis, feedback on most parts of this paper; JC: contribution and feedback to introduction and discussion; TM: supervision of all parts of this project; EM: writing of introduction; AW: data collection.

## Conflict of Interest Statement

The authors declare that the research was conducted in the absence of any commercial or financial relationships that could be construed as a potential conflict of interest.
