# Supplementary material for: Absorption in Sport: A Cross-Validation Study
Source: Front Psychol. 2017 Aug 22;8:1419. doi: 10.3389/fpsyg.2017.01419 (PMC5574407; doi:10.3389/fpsyg.2017.01419)
Supplement: Supplementary file 1 [file Table_1.doc]

Table 1

*Model-Based Estimates of Reliability and Summary of Fit Indices for all Congeneric Models*

| Factors | ρ_η_ | ρ_vc(η)_ | χ^2^ | CFI | TLI | SRMR | RMSEA | RMSEA  90% CI |
| --- | --- | --- | --- | --- | --- | --- | --- | --- |
|  | Calibration Sample | | | | | | | |
| RES | .71 | .34 | 5.116 | 1.000 | .999 | .021 | .009 | .000-.082 |
| SYN | .74 | .37 | 23.482* | .937 | .874 | .048 | .113 | .069-.160 |
| EC | .74 | .36 | 34.160* | .900 | .799 | .06 | .142 | .099-.188 |
| DI | .75 | .44 | 1.237 | 1.000 | 1.008 | .012 | .000 | .000-.101 |
| VR | .64 | .37 | 3.793 | .984 | .976 | .031 | .056 | .000-.141 |
| EA | .77 | .46 | 6.099* | .986 | .957 | .028 | .084 | .008-.164 |
|  | Validation Sample | | | | | | | |
| RES | .77 | .41 | 10.685 | .985 | .970 | .028 | .060 | .000-.111 |
| SYN | .80 | .45 | 21.590* | .963 | .926 | .038 | .103 | .061-.149 |
| EC | .71 | .33 | 5.750 | .997 | .994 | .022 | .022 | .000-.084 |
| DI | .70 | .37 | 0.100 | 1.000 | 1.026 | .004 | .000 | .000-.000 |
| VR | .72 | .46 | 7.989* | .967 | .950 | .038 | .098 | .034-.173 |
| EA | .77 | .46 | 0.536 | 1.000 | 1.014 | .03 | .000 | .000-.075 |

*Note*. **p* < .05. RES = responsive to engaging stimuli; SYN = synesthesia; EC = enhanced cognition; DI = dissociative involvement; VR = vivid reminiscence; EA = enhanced awareness. CI stands for confidence interval.

Table 2

*Examination of Fit Indices for Alternative MASC Models*

| Model | χ^2^ | *df* | CFI | TLI | SRMR | | | RMSEA | RMSEA  90% CI |  |
| --- | --- | --- | --- | --- | --- | --- | --- | --- | --- | --- |
|  | Calibration Sample | | | | | | | | | |
| M1: Single-factor model | 881.357* | 299 | .748 | .727 | | .081 | | .082 | .075-.088 |  |
| M2: Second-order model | 558.628* | 294 | .885 | .872 | | .062 | | .056 | .049-.063 |  |
| M3: Uncorrelated 6-factor model | 1173.960* | 299 | .621 | .588 | | .234 | | .100 | .094-.106 |  |
| M4a: Correlated 6-factor model | 548.368* | 284 | .885 | .869 | | .062 | | .057 | .049-.064 |  |
| M4b Correlated 6-factor model (18 items) | 207.186* | 120 | .941 | .925 | | .050 | | .050 | .038-.061 |  |
|  | Validation Sample | | | | | | | | | |
| M1: Single-factor model | 1104.612* | 299 | .705 | .681 | | .089 | .093 | | .087-.098 |  |
| M2: Second-order model | 606.949* | 294 | .885 | .872 | | .064 | .059 | | .052-.065 |  |
| M3: Uncorrelated 6-factor model | 1270.627* | 299 | .644 | .613 | | .240 | .102 | | .096-.108 |  |
| M4a: Correlated 6-factor model | 571.205* | 284 | .895 | .880 | | .059 | .057 | | .050-.064 |  |
| M4b Correlated 6-factor model (18 items) | 197.486* | 120 | .957 | .945 | | .044 | .045 | | .034-.057 |  |

*Note*. **p* < .05. Models M1 to M4a included 26 items; model M4b was based on 18 items.

Table 3

Summary of Fit Statistics for Tests of Multigroup Invariance

|  | Model | Model comparison | | χ^2^ | *df* | Δχ^2^ | Δ*df* | Statistical Significance | | CFI | | ΔCFI | |  |
| --- | --- | --- | --- | --- | --- | --- | --- | --- | --- | --- | --- | --- | --- | --- |
| M1: Configural model (no equality constraints) | | | - | 238.218 | 120 | - | - | | - | | .962 | | - | |
| M2: All item factor loadings constrained^a^ | | | 2 vs 1 | 404.827 | 258 | 166.609 | 138 | | NS | | .953 | | .009 | |
| M3: Items for RES constrained | | | 3 vs 1 | 381.262 | 243 | 143.044 | 123 | | NS | | .956 | | .006 | |
| M4: Items for RES, S constrained | | | 4 vs 1 | 385.385 | 246 | 147.167 | 126 | | *p* < .05 | | .956 | | .006 | |
| M5: Items for RES, S constrained (Item 5 freely estimated) | | | 5 vs 1 | 381.762 | 245 | 143.544 | 125 | | NS | | .957 | | .005 | |
| M6: Items for RES, S, EC constrained | | | 6 vs 1 | 383.239 | 248 | 145.021 | 128 | | NS | | .957 | | .005 | |
| M7: Items for RES, S, EC, DI constrained | | | 7 vs 1 | 395.764 | 251 | 157.546 | 131 | | *p* < .05 | | .954 | | .008 | |
| M8: Items for RES, S, EC, DI constrained (Item 11 freely estimated) | | | 8 vs 1 | 383.268 | 250 | 145.050 | 130 | | NS | | .958 | | .004 | |
| M9: Items for RES, S, EC, DI and VR constrained | | | 9 vs 1 | 387.172 | 253 | 148.954 | 133 | | NS | | .957 | | .005 | |
| M10: Items for RES, S, EC, DI VR, EA constrained | | | 10 vs 1 | 387.226 | 256 | 149.008 | 136 | | NS | | .958 | | .004 | |
| M11: Factor loadings, item intercepts constrained | | | 11 vs 1 | 425.767 | 274 | 187.549 | 154 | | NS | | .952 | | .010 | |
| M12: Factor loadings, item intercepts, and item variances constrained | | | 12 vs 1 | 463.749 | 292 | 225.531 | 172 | | *p* < .001 | | .946 | | .016 | |
| M13: Factor loadings, item intercepts, item variances, and covariances constrained | | | 13 vs 1 | 488.243 | 307 | 250.025 | 187 | | *p* < .001 | | .943 | | .019 | |

*Note*. ^a^Kline (2005) proposed that items which are fixed to 1.0 cannot be examined for invariance. Therefore, these items were freed and the latent parent variables were fixed to 1.0.

Table 4

*Testing for Latent Mean Differences across Validation and Calibration Samples*

| MASC | Mean  Estimate | S.E. | C.R. | *p* |
| --- | --- | --- | --- | --- |
| 1. Responsiveness to engaging stimuli | .074 | .093 | 0.792 | .428 |
| 2. Synesthesia | .080 | .092 | 0.866 | .387 |
| 3. Enhanced cognition | .178 | .103 | 1.732 | .083 |
| 4. Dissociative involvement | .227 | .101 | 2.247 | .025 |
| 5. Vivid reminiscence | .017 | .098 | 0.177 | .860 |
| 6. Enhanced awareness | -.020 | .096 | -0.206 | .837 |

*Note*. **p* < .05; S.E. = Standard Error; C.R. = Critical Ratio. Dividing the estimate of the mean

by the estimate of its standard error provides a z-score above or below zero. Critical ratios

above or below 1.96 are significantly different from zero (Arbuckle, 2012).

Table 5

*Items with Standardized Factor Loadings (FL) for the Final 18-item First-Order MASC Model*

| No | Items | Factor | *FL* | *IU* |
| --- | --- | --- | --- | --- |
| 1. | I can be deeply moved by an outstanding sport performance. | RES | .72 | .51 |
| 2. | Watching a great game or performer in my sport stimulates my imagination. | RES | .84 | .71 |
| 3. | I can be greatly moved by elegant or graceful sport moves or strokes. | RES | .67 | .44 |
| 4. | Sometimes the sounds of my sport sound like music to me. | SYN | .73 | .54 |
| 5. | Some of my most vivid memories of my sport are called to my mind by scents and smells. | SYN | .59 | .35 |
| 6. | The specific sounds that occur in my sport can be so fascinating to me that I can just go on listening to them. | SYN | .75 | .57 |
| 7. | I often have "physical memories"; for example, after I completed a sport session, I may still feel as if I am performing. | EC | .60 | .37 |
| 8. | While participating in my sport, my thoughts often don't occur as words, but as visual images. | EC | .65 | .33 |
| 9. | While participating in my sport, sometimes thoughts and images come to me without the slightest effort on my part. | EC | .62 | .38 |
| 10. | While doing my sport, I can get so caught up in what I am doing that I don’t notice anything else. | DI | .79 | .62 |
| 11. | While participating in my sport, it is sometimes possible for me to be completely immersed in the situation and to feel as if my whole state of consciousness has somehow been temporarily altered. | DI | .68 | .47 |
| 12. | I am able to wander off into my own thoughts, while I am doing a routine task in my sport, and then find a little later that I have completed it. | DI | .45 | .20 |
| 13. | Doing my sport, sometimes I feel and experience things as I did when I was younger. | VR | .62 | .38 |
| 14. | If I stare at a picture portraying a situation in my sport and then look away from it, I can sometimes "see" an image of the picture, almost as if I were still looking at it. | VR | .74 | .54 |
| 15. | I can sometimes recollect certain past experiences in my sport with such clarity and vividness that it is like living them again or almost so. | VR | .68 | .46 |
| 16. | While participating in sport, I sometimes "step outside" my usual self and experience an entirely different state of being. | EA | .71 | .50 |
| 17. | While doing my sport, sometimes I experience things as if they were doubly real. | EA | .77 | .60 |
| 18. | In my sport, things that might seem meaningless to others often make sense to me. | EA | .60 | .36 |

*Note*. FL = factor loading; IU = item uniqueness. RES = responsive to engaging stimuli; SYN = synesthesia; EC = enhanced cognition;

DI = dissociative involvement; VR = vivid reminiscence; EA = enhanced awareness.

Table 6

*Correlations between the MASC and DFS-2 Factors*

|  | Calibration Sample (*n* = 287) | | | | | |  | Validation Sample (*n* = 308) | | | | | |
| --- | --- | --- | --- | --- | --- | --- | --- | --- | --- | --- | --- | --- | --- |
| DFS-2 | RES | SYN | EC | DI | VR | EA |  | RES | SYN | EC | DI | VR | EA |
| Challenge-Skills Balance | .25** | .12* | .23** | .32** | .24** | .30** |  | .14* | .06 | .06 | .23** | .16** | .19** |
| Action-Awareness Merging | 0.11 | .06 | .17** | .29** | .18** | .21** |  | .14* | .04 | .21** | .34** | .24** | .28** |
| Clear Goals | .25** | .10 | .11 | .22** | .18** | .25** |  | .13* | .16** | .19** | .24** | .28** | .32** |
| Unambiguous Feedback | .22** | -.06 | .12* | .13* | .17** | .19** |  | .14* | .02 | .18** | .16** | .18** | .25** |
| Concentration on the Task at Hand | .21** | .17** | .14* | .31** | .21** | .30** |  | .16** | .07 | .12* | .27** | .20** | .30** |
| Sense of Control | .01 | .04 | .07 | .15* | .13* | .21** |  | .22** | .17** | .19** | .28** | .24** | .31** |
| Loss of Self-Consciousness | .02 | .09 | .04 | .12* | .05 | .14* |  | .12* | .02 | .03 | .13* | .14* | .14* |
| Time Transformation | .23** | .16** | .17** | .34** | .18** | .30** |  | .13* | .09 | .19** | .30** | .17** | .30** |
| Autotelic Experience | .35** | .15* | .18** | .26** | .22** | .35** |  | .27** | .26** | .35** | .35** | .34** | .41** |

*Note*. **p* < .05; ***p* < .01. RES = responsive to engaging stimuli; SYN = synesthesia; EC = enhanced cognition; DI = dissociative involvement; VR = vivid reminiscence; EA = enhanced awareness.
